# Towards a sustainable astronomical data infrastructure: Optimising linking data from the Rucio datalake to the users areas within the SKA Regional Centres Network

**DOI:** 10.12688/openreseurope.22118.3

**Published:** 2026-08-10

**Authors:** Manuel Parra-Royón, Julián Garrido-Sánchez, Susana Sánchez-Expósito, Laura Darriba-Pol, Jesús Sánchez-Castañeda, M. Ángeles Mendoza, Jeremy Coles, Sean McConkey, Rohini Joshi, Rob Barnsley, Jesús Salgado, Lourdes Verdes-Montenegro

**Affiliations:** 1 Extragalactic Astronomy, Instituto de Astrofisica de Andalucia, Granada, Andalusia, 18008, Spain; 2 Battcock Centre for Experimental Astrophysics, University of Cambridge Department of Applied Mathematics and Theoretical Physics, Cambridge, England, CB3 0HE, UK; 3 Institute for Data Science, Hochschule fur Technik FHNW, Windisch, Aargau, 5210, Switzerland; 4 SKA Organisation, Macclesfield, England, SK11 9FT, UK

**Keywords:** Data preparation, Federated data infrastructure, SKAO, Mount propagation, Storage orchestration, Cloud-native architectures, Green Computing

## Abstract

The distributed architecture of the SKA Regional Centre Network (SRCNet) aims to provide scientific communities worldwide with efficient computational and storage resources to exploit the massive data volumes produced by the SKA Observatory (SKAO). Given the amount of SKAO data, traditional data management paradigms — where data is transferred to computational resources— are no longer feasible. Instead, computational workflows must increasingly be relocated closer to data storage locations, emphasizing efficient data access strategies and avoiding unnecessary duplication or redundancy. In this context, we present PrepareData, a modular and extensible data delivery service developed within SRCNet prototyping activities. Our proposal for this service addresses the critical challenge of redundant data transfers and duplication at both node and user levels by enabling seamless delivery of requested datasets from local Rucio Storage Elements (RSEs) directly into users’ working environments. PrepareData operates as a local service within each SRCNet node and it is integrated into a broader ecosystem of federated services. Specifically, we designed and evaluated two distinct yet complementary implementations to avoid unnecessary data duplication and to enable a dynamic data bridge between the RSEs and the user storage areas, through: (1) a filesystem-based solution leveraging CephFS, which uses shared filesystem mount points and bind mounts to ensure consistent and immediate data availability of the data across computational nodes, and (2) a Kubernetes model using Persistent Volumes and Persistent Volume Claims, dynamically injecting data into a user’s areas. To tackle this work, we detail the architectural design and development, the technical implementation, the integration of both solutions with science-enabling tools such as JupyterHub, CARTA, or virtually any application, and, finally, we present a trade-off analysis based on operational preparation times, data-access implications, and orchestration overheads. This contribution provides a scalable and sustainable blueprint for data delivery in federated scientific infrastructures, supporting the broader goals of green computing and efficient resource utilisation.

## Plain Language Summary

Modern scientific projects such as the Square Kilometre Array Observatory (SKAO) produce extremely large amounts of data — far more than traditional research systems have handled before. Scientists around the world need efficient ways to access and work with these vast datasets without unnecessary delays or wasteful copying of information. In the SKA Regional Centre Network (SRCNet), data are stored in distributed storage systems. However, these storage systems are not directly visible to the software tools scientists use for analysis, such as interactive notebooks, visualisation platforms, or specialised processing services. To make the data usable, it must be moved or linked into the scientists’ working environments. Our research focuses on a service called *PrepareData*, designed to manage this delivery process in a way that is both fast and resource-efficient. We describe and test different technical methods for exposing data to users, including techniques that avoid repeatedly copying large files. We ran experiments to measure how fast and scalable each method is under realistic conditions. The results show that by linking data instead of copying it, *PrepareData* can reduce delays and lower the burden on storage systems. This leads to better performance for scientists and less wasted computing and storage resources. These improvements are especially important for major international science projects, where efficient data delivery can accelerate research and reduce environmental impact.

## 1. Introduction

The Square Kilometre Array Observatory ^1^ (SKAO) and its distributed network of SKA Regional Centres [1] (SRCNet) face unprecedented challenges in managing data scale and movement, with SKA data volumes expected to reach exabyte levels. ^2^ The SKAO Data Lake is orchestrated with Rucio, ^3^ a policy-driven data management system that automates replication, placement, and lifecycle control across heterogeneous storage infrastructures. In Rucio, data are persisted in *Rucio Storage Elements* (RSEs), logical storage endpoints that abstract diverse backends (e.g., Ceph, ^4^ dCache, ^5^ EOS, ^6^ and cloud object stores) under a unified namespace. Crucially, replicating every dataset to *all* RSEs is infeasible at SKA scale due to storage, bandwidth, and operational costs. ^7^ Instead, datasets must be placed selectively on a minimal subset of SRCs (via their local RSEs) to balance scientific demand against resource constraints.

Access to these data and the associated compute capacity is provided, among others, through the SRCNet *Science Gateway*, which integrates data discovery, resources selection, and orchestration of data processing across SRC nodes. ^8^ Within representative SRCNet use cases, ^9^ scientists can launch science services—such as Jupyter Notebooks or visualisation tools — on the infrastructure where the data physically reside—typically the local RSE at a chosen SRC. However, because RSEs expose a storage abstraction optimised for global data management rather than user-facing run-times, datasets residing in an RSE are not directly visible or consumable by end-user applications. Local SRC nodes therefore require a mechanism to *deliver* data from the RSE into user workspaces (e.g., home directories or service-attached paths) in a way that is both functional and efficient for analysis, visualisation, and processing tools. Data infrastructures support several access patterns that do not expose complete datasets through filesystem paths. These include object-storage-native interfaces, API-mediated access, pre-signed URLs, and subsetting or cutout services that return only the data region required by a particular workflow.

A straightforward mechanism for filesystem-oriented data-delivery strategies, is to copy the requested data from the local RSE into the user area prior to execution. While operationally simple, this approach incurs duplication overheads, can introduce non-negligible preparation latency for large files, and exerts sustained pressure on user quotas and shared storage—particularly under concurrent demand. Although adequate for testing or low-volume scenarios, it is unlikely to remain viable at the operational scale foreseen for SRCNet.

SRCNet prototyping teams developed *PrepareData*, a service designed to deliver data from the RSE to the user workspaces, initially based on the copy strategy but open to include other additional strategies. In this context, we have designed and developed a local data-delivery strategy that avoids wholesale copying by *exposing* RSE-resident datasets within user workspaces. First, *operating-system–native* mechanisms (symbolic links and mount --bind) enable efficient linking from the RSE into user workspaces-visible paths. Second, *Kubernetes-native* mechanisms (based on Persistent Volumes and Persistent Volume Claims, PVs/PVCs) allow per-user dataset attachment within orchestrated services. Together, these approaches support both standalone services (e.g., SLURM-based workflows and other non-orchestrated applications) and Kubernetes-hosted environments (e.g., CANFAR, ^10^ visualisation tools, Jupyter Notebooks or virtually any Kubernetes service) without duplicating data. This design is shaped by the need to accommodate heterogeneous computing and services, and data storage environments, aiming to minimize redundant transfers within the local SRCNet nodes and fostering the adoption of mechanisms aligned with green computing.

This work addresses the problem of efficiently delivering datasets stored in local RSEs to user workspaces within SRCNet without unnecessary data duplication. We focus exclusively on filesystem-oriented data-delivery strategies, while alternative access patterns are left for future investigation. We develop alternative delivery strategies that allow direct access to RSE-resident data while maintaining performance and scalability. The main contributions are: (i) the design of operating-system–native mechanisms and Kubernetes-native approaches (PVs/PVCs) for dataset exposure; (ii) their integration into the PrepareData service to support heterogeneous execution environments; and (iii) their assessment as an alternative to traditional copy-based methods, reducing storage and redundant data movement. The evaluation examines functional deployment, operational characteristics, and the suitability of each approach for different execution scenarios, rather than establishing a general performance ranking or providing comprehensive security validation, policy enforcement, or lifecycle management.

These resulting mechanisms enable an end-to-end workflow in which scientists can select an SRC through the Science Gateway, run analyses close to the data at the local RSE, and seamlessly access those datasets from their runtime environments. By combining these selective data placements across RSEs with local delivery mechanisms that eliminate redundancy, *PrepareData* bridges the gap between global data management and user-centric execution, providing a scalable and operationally efficient solution to consider given the performance requirements of SKA-scale science.

This paper is organised as follows. Section 2 provides an overview of existing approaches for user data provisioning within large-scale scientific infrastructures, high-lighting current limitations and motivating factors. Section 3 presents the architectural landscape of the SRCNet, outlining the operational and technical requirements that drive the need for the *PrepareData* service. The development, functional components and integration of the proposed solution are described in Section 4, including the data delivery strategies and modular architecture. Test experiments and comparative trade-off results are presented in Section 5. Finally, Section 6 discusses future directions, including support for hybrid deployment models and interoperability across distributed RSEs.

## 2. Related work

The delivery of large-scale scientific datasets to users is a recurring challenge in Big Science platforms such as SKAO, ^11^ LOFAR, ^12^ and other observatories. A foundational contribution in this domain in 13, presents a scalable data delivery framework for astronomical observatories. Their system implements automated staging, user-aware transfer policies, and metadata tracking while reducing redundant copying—principles that align closely with the goals of *PrepareData.*

Several large-scale scientific collaborations have developed specialized data management systems to address the challenges of data duplication and efficient user access. For the ATLAS experiment at CERN, Rucio ^3^ was developed to manage over a billion files across more than 120 data centres, facilitating policy-driven data placement and minimizing unnecessary data replication. In ATLAS, data were organised into a very large number of relatively small files, a granularity well suited to replication across distributed storage. By contrast, SKAO products will consist of much larger files (≈ TB), making replication to every RSE prohibitively costly in both storage and bandwidth. Another approach working at CERN based on EOS ^5^ storage system, combined with CERNBox, offers users synchronized access to their data across various platforms, reducing the need for redundant data transfers. Another example is the dCache service, used by DESY [2] and Fermilab [3], where it provides a unified virtual filesystem that allows seamless access to distributed datasets without the need for multiple copies ^15^ but within the context of High Energy Physics. For instance, in another science context ^16^ proposes a memory-based caching mechanism that mitigates file system contention and accelerates data availability using in-memory staging on compute nodes. Similarly ^17^ introduces a scalable service for petascale systems, enabling intermediate staging from compute nodes to dedicated staging nodes before final persistence.

Additional other contributions address complementary needs in data workflows. For instance, ^18^ explores the seamless integration of interactive Jupyter workflows with distributed datasets via shared file systems, while in 19 evaluates the performance characteristics of different storage backends in large-scale image analysis pipelines, highlighting trade-offs between latency, throughput, and data isolation.

Beyond domain-specific platforms, the broader literature emphasizes key requirements for efficient data access in scientific infrastructures. Deduplication has been proposed as a critical energy-saving and scalability mechanism in cloud and distributed storage environments. Khan *et al.* ^20^ describe a fault-tolerant, cluster-wide deduplication implementation in Ceph environments, showing high robustness and near-optimal space savings with minimal performance impact. Such approaches directly support the green-computing vision by reducing redundancies and operational energy usage. Furthermore, the energy-efficient storage architecture proposed in 21 uses online deduplication for VM image stores, reporting a reduction of up to 66% in energy consumption while preserving performance, an indication of how data preparation strategies that avoid duplication can contribute to sustainability.

At the orchestration level, ^22^ propose a time-driven data placement strategy that combines edge and cloud computing to minimize data transfer latency, adapting the placement dynamically based on bandwidth and storage constraints. While their context differs, the principle of bringing computation close to data resonates with the “move computation to data” model adopted in SRCNet.

Within green computing research, general principles like reuse, reduction, and support —Green Data Mining— have emerged as design tenets for sustainable data workflows. ^23^ These works illustrate the importance of reducing data duplication, supporting heterogeneous back-end infrastructures, enabling lifecycle-aware access control, and integrating robust user authentication and authorisation. They provide strong motivations and justification for the architecture behind *PrepareData*, which couples RESTful API interaction, asynchronous state management, and modular backing implementations to deliver efficient, scalable, and environmentally-conscious data delivery in distributed scientific environments like the SRCNet.

## 3. Problem statement

While the SRCNet has 16 member countries, its initial phase is focused on the more advanced initiatives from nine countries, notably including the Spanish SRC. ^24^ Each initative is setting up an SRC that will collectively deliver access to around 700 PB of data per year to the global SKAO user scientist community. As the volume and complexity of scientific data continue to grow across distributed research infrastructures, ensuring efficient, scalable, and user-transparent data delivery mechanisms has become a critical challenge.

An initial phase of the work focused on enabling user access to data through the deployment of a Rucio-based datalake and the development of connectors for both interactive tools and batch processing environments. This provided a preliminary solution for transferring data from the RSE to the user workspace, but also exposed several limitations related to data distribution and repeated data movement to the local sessions. To address these issues, subsequent efforts concentrated on integrating the RSE, the user area and a linking mechanism capable of exposing datasets without redundant transfers. This line of work ultimately led to the design and implementation of the *PrepareData* system. In this context, the ability to prepare and expose data to end users—while minimizing duplication, latency, and resource contention—is essential for interactive and high-performance scientific workflows in next generation observatories in line with SKAO and the SRCNet.

**Figure 1.**
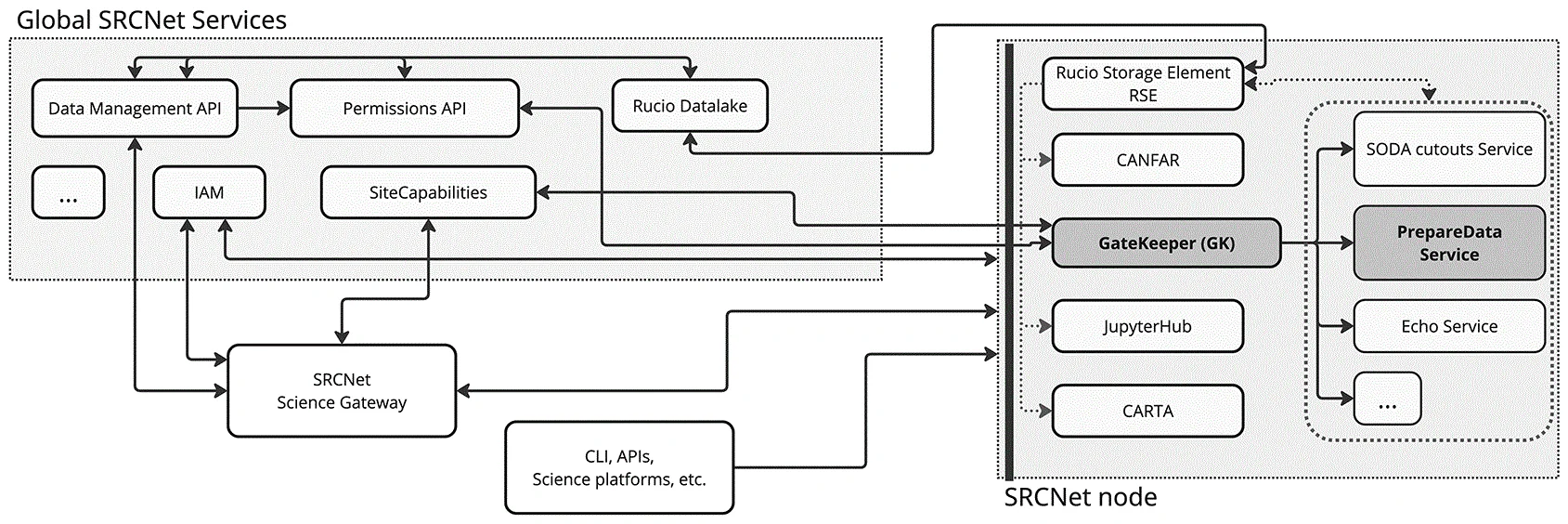
Architecture of the SRCNet services ecosystem, distinguishing between global services (SRCNet Global Services) and local services (SRCNet nodes), together with their dependencies. The diagram highlights the mandatory services required at SRC nodes and the specific integration of the PrepareData service. Access to PrepareData and other data-related services is protected and authorised through the GK component.

Figure 1 illustrates the architectural foundation of the SRCNet ecosystem, which comprises multiple SRC nodes coordinated by the Global SRCNet services. Each SRC node hosts a range of scientific services and resources that are accessible both through a Science Gateway and independently. In this figure are highlighted several key components involved in managing data and orchestrating user workflows, including the DM-API (SRCNet Data Management API), SC-API (SRCNet Site Capabilities API), SRCNet Permissions API, and then local services exposed by the SRC nodes through the SRCNet GateKeeper [4] (GK)—such as the *PrepareData* service or the SODA ^25^ cutout service—, and other top level SRCNet local services like CANFAR [5], JupyterHub or CARTA, ^26^ an image visualisation and analysis tool, among others. *PrepareData* and other dependent services need GK to: (1) validate that the user has the necessary permissions to access a given dataset and its corresponding Rucio namespace within the SRCNet node; and (2) verify that the requested service is available and operational within the selected SRC node.

**Figure 2.**
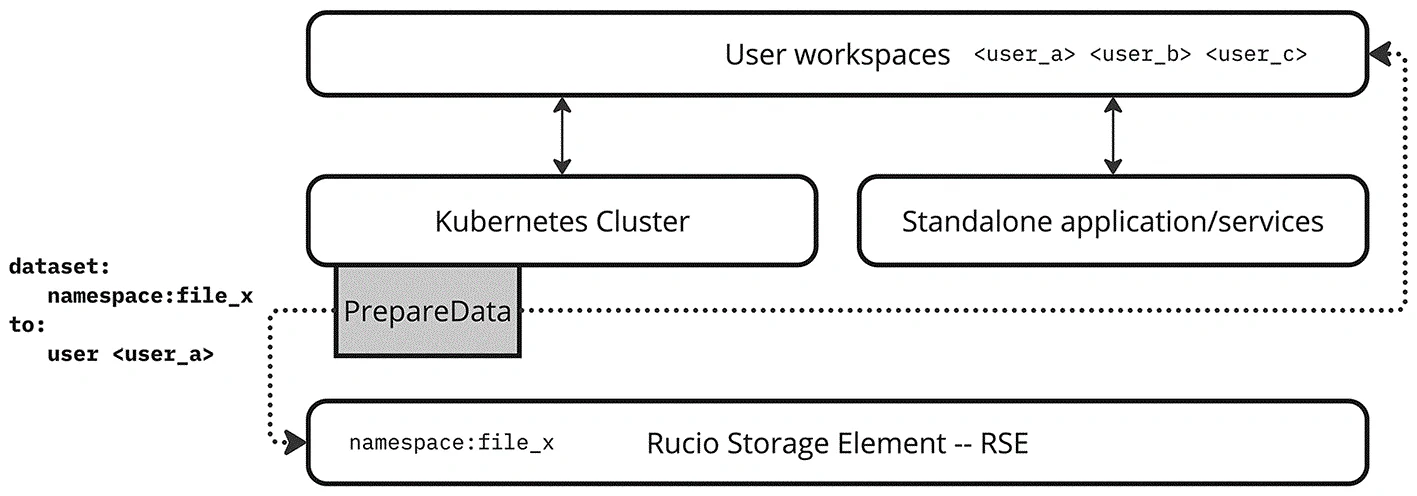
*PrepareData* acts as a bridge between the local RSE of the SRC and user storage areas, enabling access to data in user areasthat are accessible from services running both standalone and on Kubernetes. Here, *PrepareData* runs on Kubernetes.

One of the central challenges within the current SRCNet architecture lies in efficiently delivering scientific data from the local Rucio Storage Element (RSE) of each SRC node to the user’s working environment. In this federated infrastructure, user workspaces are provisioned locally within each SRC, yet the RSEs are not directly accessible or visible to users due to their design as controlled backend storage end-points. Consequently, an intermediary mechanism is required to make these datasets available within each user’s environment, enabling seamless use by scientific tools such as JupyterHub, CANFAR, or CARTA. The most direct way to achieve this delivery is to copy the required datasets from the RSE into the user’s workspace before the analysis begins. This approach ensures native accessibility from user applications but introduces significant limitations, including data duplication, latency from repeated transfers, and inefficient utilisation of local storage capacity. In large-scale scientific environments such as the SRCNet, where multiple users may request overlapping datasets, this model rapidly becomes unsustainable in terms of performance, cost, and scalability.

Given this context, *PrepareData* is conceived as the service responsible for bridging the gap between data stored in the local RSE and the user-accessible workspace (see Figure 2). The service must not only ensure data availability and accessibility for the user but also do so in a way that respects the architectural heterogeneity of the SRCNet. This heterogeneity includes both standalone services (e.g., batch systems such as SLURM or interactive environments) and Kubernetes-based orchestrated deployments, as well as heterogeneous backend storage systems ranging from POSIX filesystems to Ceph- or xrootd, dCache-based RSEs.

Under these conditions, simply extending the default copy-based behaviour of *PrepareData* is not sufficient. New delivery strategies are needed that avoid unnecessary local data movement, instead leveraging operating-system and orchestration-level capabilities to expose RSE data efficiently within user environments. These strategies must preserve user transparency—data appearing natively within the user’s workspace—, and ensure authorised access to the data, while optimising system efficiency by reducing bandwidth consumption, data duplication, and I/O overhead across SRC nodes.

This work addresses these challenges by presenting both currently deployed operational solutions and new approaches under development within the SRCNet prototyping framework. The proposed mechanisms target two main scenarios: (i) services operating in standalone mode and (ii) services deployed within Kubernetes clusters. For the latter, we focus on two complementary data delivery strategies—mount propagation and the use of Persistent Volumes and Persistent Volume Claims (PVs/PVCs)—which are designed to minimise duplication, reduce data-access latency, and establish a sustainable, resource-efficient model for user data provisioning in distributed scientific environment.

## 4. Development of PrepareData

**Figure 3.**
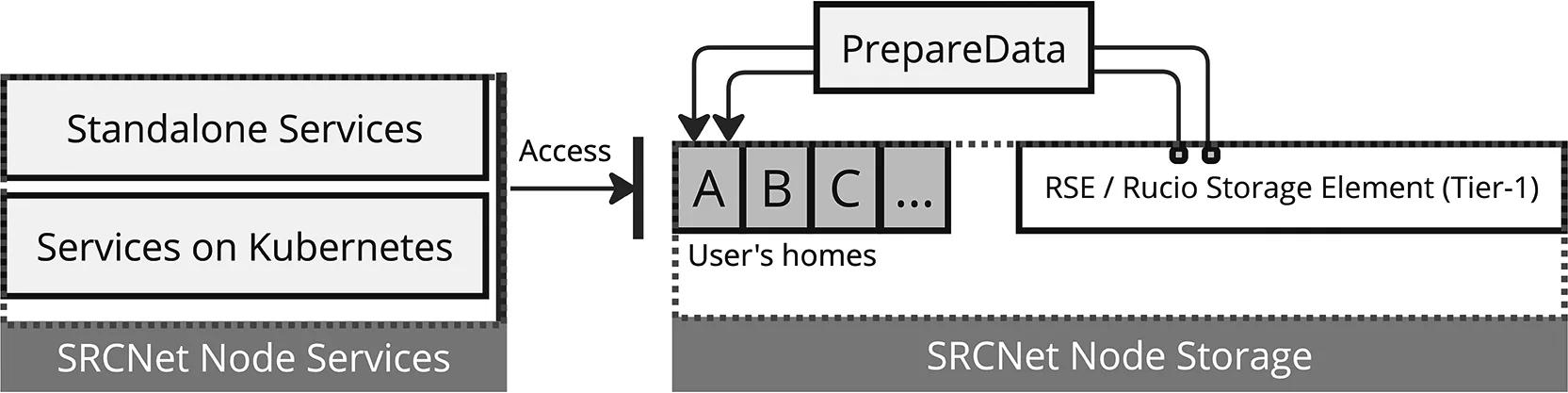
This diagram illustrates how services in local SRCNet nodes support both standalone and Kubernetes based deployments, and how *PrepareData* acts as an intermediary between the Rucio RSE (local Rucio storage) and the user workspace (user homes) within the SRCNet node storage.

In the current distributed infrastructure of the SRCNet, user home directories are currently isolated per SRC site, requiring a mechanism to expose requested data locally (see Figure 3). This functionality must abstract the underlying heterogeneity of storage back-ends and it provides a uniform mechanism for dataset exposure. It must support two primary usage scenarios: services executed within orchestrated environments (e.g., Kubernetes) and standalone applications running directly on the hosts system. The solution must also ensure that data access complies with user permissions and that the exposed datasets are correctly mapped to the corresponding user workspace without duplicating data unnecessarily. Among the constraints, the system must operate under limited storage quotas, maintain low latency in access times, and guarantee isolation between user sessions. At the same time, the service needs to interoperate with other SRCNet services through agreed interfaces in order to maintain independence among the SRCNet services.

**Figure 4.**
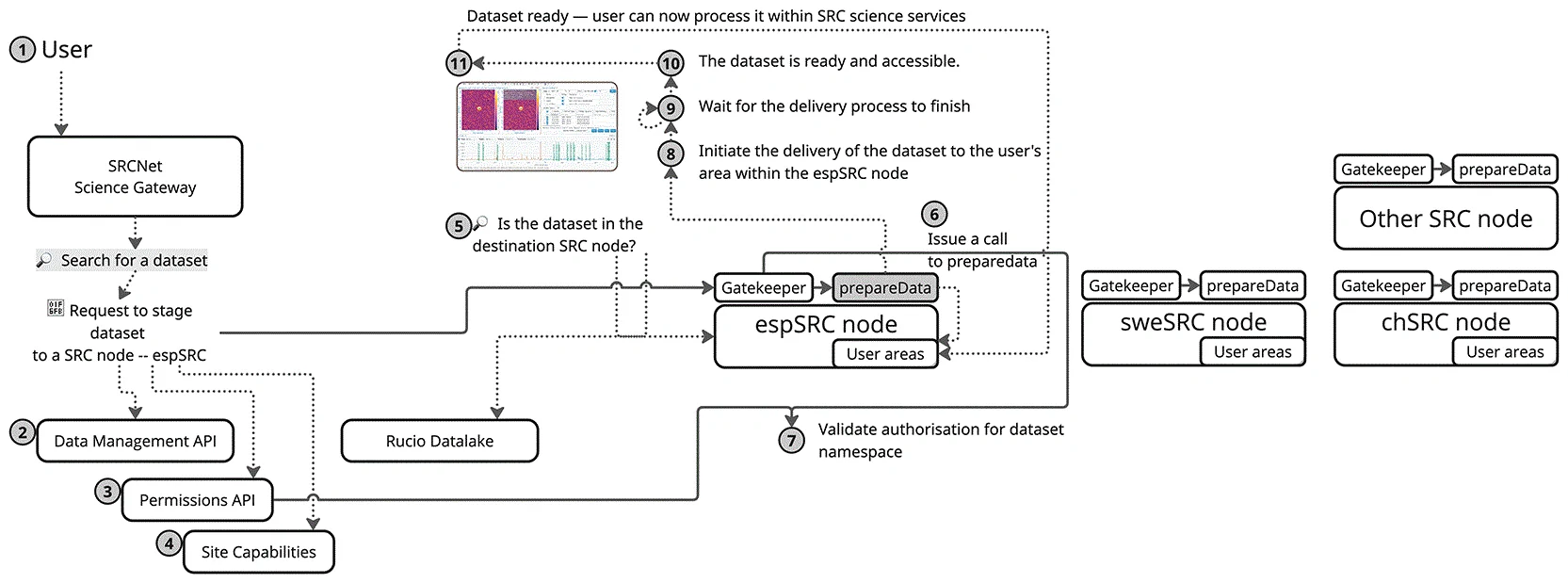
This diagram shows the workflow of operations and services involved in this specific use case, from the moment a scientist accesses the SRCNet Science Gateway until the requested data are made available in their user workspace.

Figure 4 illustrates a sequence of operations of a use case triggered when a dataset is staged to a SRC. In the current implementation, users must explicitly initiate the staging process and specify where data should be transferred. However, the final design aims to make this entirely transparent to the user: the system should automatically determine the appropriate destination without requiring manual intervention. The workflow for the current implementation is composed of the following steps:

1. An user initiates a request to prepare a dataset at a specific SRC site.
2. A preliminary check is performed to determine whether the dataset is already available at site. If not, a staging request is issued to transfer the dataset asynchronously to the corresponding RSE to access the selected dataset.
3. Metadata and site-specific information are retrieved from the SRCNet registry and information services (SRCNet Site Capabilities).
4. If the dataset is not present in the RSE, a request is made to trigger the asynchronous data transfer.
5. If the dataset is already available, a request is issued to the *PrepareData* service deployed at the SRCNet node.
6. Authorisation mechanisms validate that the user has the required privileges to execute data preparation actions at the site.
7. The *PrepareData* service is invoked, launching a local job that places the dataset into the user’s area — typically via copy, symbolic links, mount propagation, or PVs/PVCs.
8. The system monitors the execution status of the preparation job and waits for it to reach a terminal state, typically READY.
9. Once the job is completed successfully, the dataset becomes accessible within the user’s area in the selected SRCNet node.
10. Finally, the user opens a science-enabling application (CARTA, JupyterHub, etc.) to explore/use the requested data.

**Figure 5.**
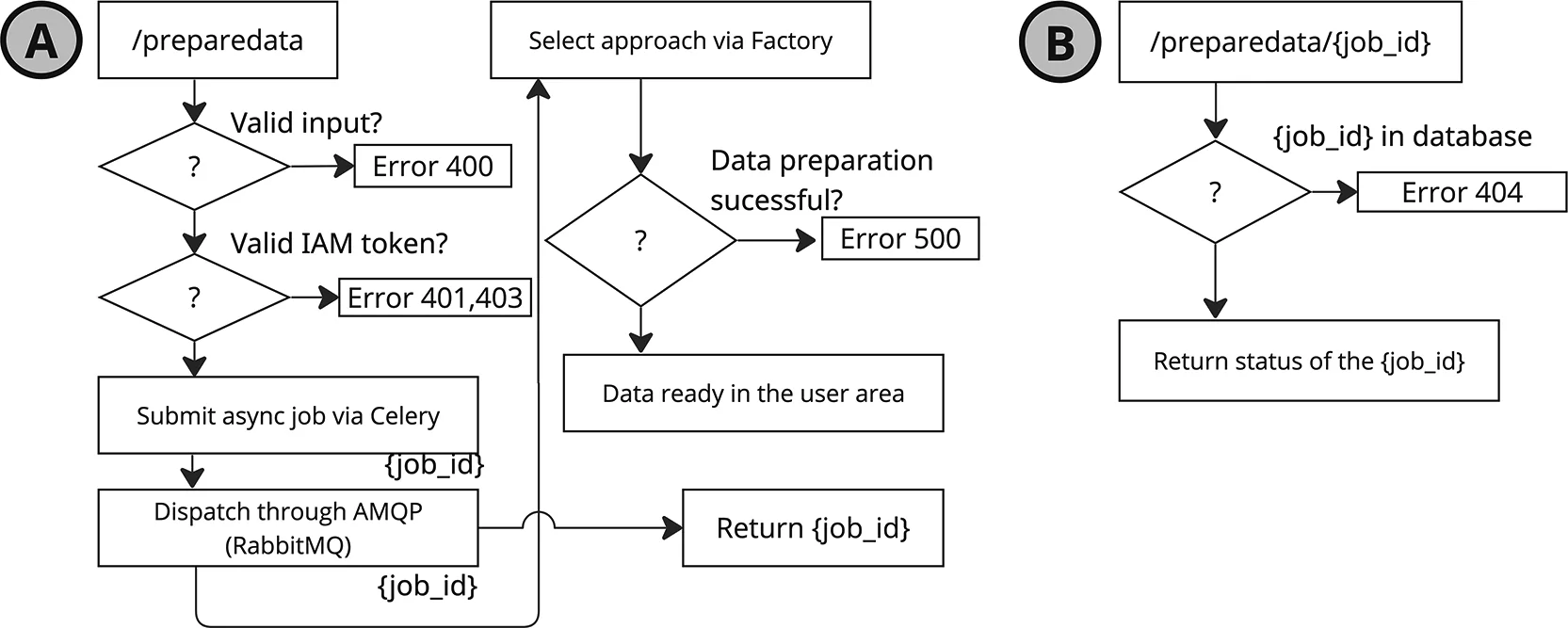
Diagram of the two *PrepareData* calls: ( **A**) the initial request with parameters, and ( **B**) the status query to check the state of the data preparation job.

**Figure 6.**
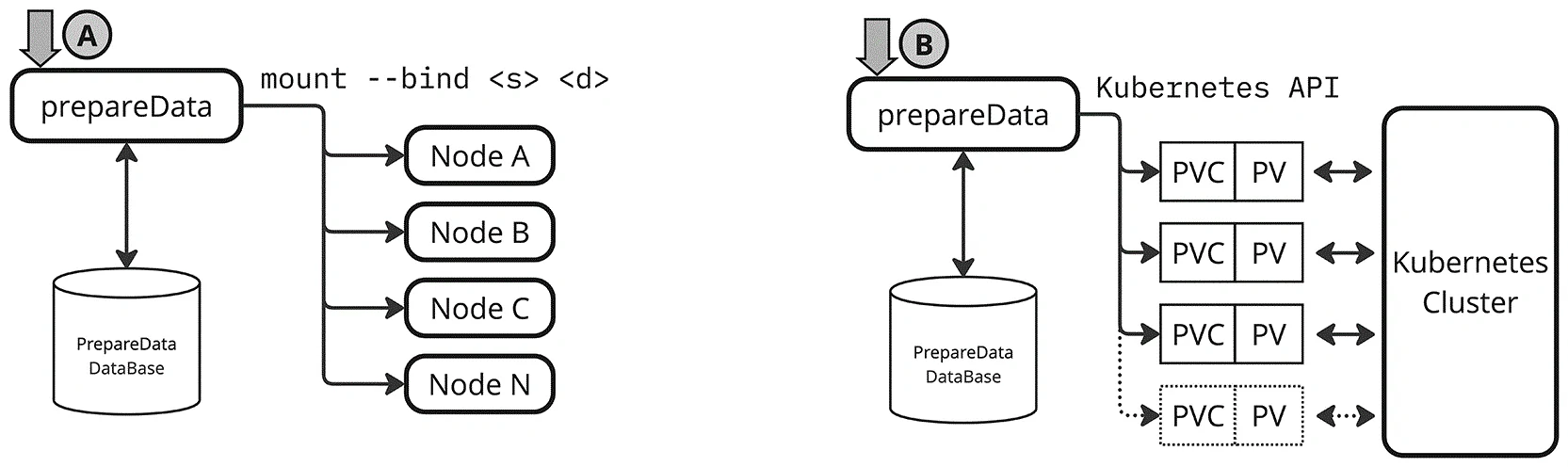
Scheme of operation of mount and PV/PVCs approaches.

In order to ensure consistent interfaces across all SRC nodes and enable seam-less integration with other components of the SRCNet ecosystem, the *PrepareData* functionality must expose a Web service with a standardized API. This is achieved through a RESTful API, which provides a homogeneous interaction layer regardless of the underlying infrastructure. The API is designed to receive as input an OIDC token –used for user authentication and authorisation– as part of the HTTP Authorization Bearer header, along with a payload containing a list of datasets to prepare for the local user. Each dataset entry specifies the logical dataset name, the corresponding path in the local RSE, and the user-relative mount point (typically under the user’s home directory). The API specification has been formally defined using the OpenAPI standard. ^27^ An example API call by using curl command is illustrated below in the next listing:

curl -X ‘POST’ \
‘http://localhost:8000‘\
-H ‘accept: application/json’ \
-H ‘Content-Type: application/json’ \
-H ‘Authorization: Bearer $SKA_TOKEN$’ \
-d ‘[
[
“testing:galactic_centre.fits”,

“testing/84/1c/galactic_centre.fits”,
“./testing”
]
]’



In this request, the service will prepare the dataset galactic centre.fits, identified logically under the testing namespace, whose actual location in the RSE is: testing/84/1c/galactic_centre.fits. The file will be made accessible within the user’s area, allocated in the subdirectory ./testing relative to the user home. The invocation of the *PrepareData* functionality is designed to be intermediate by GK, as illustrated in Figure 4. This architectural decision ensures consistency in access control and direct invocation of the *PrepareData* API is only permitted in local testing environments; in production scenarios, all external requests are routed through the GK.

### 4.1 Service implementation

The *PrepareData* service has been implemented in Python, leveraging the FastAPI ^28^ framework for building a high-performance RESTful web interface, and served using the uvicorn [6] ASGI server. To support multiple data preparation implementations in a modular and extensible way (see details in 4.2), *PrepareData* adopted the object factory design pattern, which allows us to integrate our custom implementations seamlessly. Since the delivery of data from the RSE to the user’s area is not instantaneous and may vary significantly depending on the specific implementation used (e.g., linking vs. copying), the *PrepareData* service implements an asynchronous processing –without blocking subsequent operations– model as depicted in the Figure 5.

To handle asynchronous tasks, the service relies on Celery [7], a distributed task queue, which enables the delegation of long-running or delayed tasks (see Figure 5). Upon initiation of a data preparation request, a unique preparation identifier ( job_id) is generated and returned to the client. This identifier can be used in subsequent API calls to query the status of the operation.

The lifecycle of a data preparation request is captured through a finite set of states, where in the *Pending* state, the preparation task has been accepted and is currently being processed. Once the requested data have been successfully made available in the user’s home directory, the process enters the *Completed* state, indicating that the data are ready to be used by a scientific service. Finally, in the *Error* state, the data preparation has failed due to several conditions: a) the service may be unable to stage or link the dataset from the local RSE to the user’s area; b) the requested dataset may no longer exist in the RSE due to expiration or policy-driven deletion; c) the user’s home directory may be unavailable because no session has been initiated; d) or there may be insufficient storage space to accommodate the requested data.

### 4.2 Data preparation implementations

The *PrepareData* service offers a flexible mechanism for integrating multiple implementations within its codebase based on *objects factory.* This design principle allows for the modular inclusion of different implementations without modifying the core service logic. As a result, each SRC could deploy one or several implementations tailored to its available infrastructure and the needs of the services it exposes.

***4.2.1 Copy-based delivery***

The copy-based approach represents the most time-consuming scenario among the supported data preparation strategies. It is primarily designed as a baseline implementation to validate the operational correctness of the *PrepareData* service. In this mode, data are physically copied from the local RSE to the user’s area.

Its current implementation leverages standard operating system call and wrappers (e.g., cp) to perform file transfers. This approach does not maintain any internal registry or tracking metadata about which files were delivered to which user, nor the context of the data preparation job. As a result, this prevents *PrepareData* from executing automated garbage collection or lifecycle control (e.g., revocation, expiration) over the prepared datasets.

Additionally, this method is not using any caching or deduplication logic. If a dataset is already available either at the RSE or even within a user’s home directory, the copy is performed again without verification. Consequently, repeated requests for the same dataset by the same or different users will result in multiple independent copies in their respective user areas. This behavior may lead to unnecessary storage consumption and redundant data replication, which is unsustainable at scale. A more optimal implementation of copy-based delivery could have been achieved by introducing, for example, mechanisms to detect and avoid redundant copies when the dataset is already present at the target RSE; however, such enhancements were considered out of scope for this work.

The total preparation time includes two distinct phases: (i) a potential staging period, if the dataset is not already present in the local RSE, and (ii) the copy time from the RSE mount point to the user’s home directory.

***4.2.2 Mount bind and mount propagation***

This approach leverages a Linux kernel feature known as mount --bind, which allows a file or directory that is already part of the filesystem to be remounted at another location. Unlike symbolic links, mount --bind operate at the kernel level and **effectively** mirror a directory tree from one path to another within the same namespace, preserving permissions and access controls.

In the context of *PrepareData*, this mechanism enables dynamic linking of files located within the RSE directly into a user’s area, which may reside on a separate volume or filesystem. With all, this method ensures data encapsulation, as users are only granted access to the files within their designated home directories, even though the underlying storage is shared. It also prevents privilege escalation or unauthorized browsing of unrelated directories.

However, this approach requires root-level privileges (or equivalent capabilities) to execute mount –bind operations on the hosts. Furthermore, since Kubernetes workloads are typically distributed across multiple nodes, the bind operation must be consistently applied on all nodes where the target pod might be scheduled. To address this, *PrepareData* uses mountPropagation: HostToContainer [8] to propagate the mounted path from the host into the containerized workload in Kubernetes. Figure 6 presents the high-level architecture of the proposed solution. The diagram illustrates how the *PrepareData* service is integrated with a backend database responsible for managing metadata associated with each data preparation request.

For each request, the implementation registers the job_id, the target user, the original dataset path within the RSE, and the destination path within the user’s area. In addition to this core metadata, the database stores timestamps such as creation and expiration among other. This enables future lifecycle management features, such as automated permissions revocation or unlinking of datasets once their access window expires.

***4.2.3 Persistent volume-based linking***

Given that a significant portion of the scientific services deployed in the SRCNet architecture are or will be deployed on Kubernetes, *PrepareData* have also support native data provisioning mechanisms compatible with the orchestrator storage model. Kubernetes provides a storage abstraction through PVs and PVCs. A PV represents a piece of storage in the cluster, provisioned either statically or dynamically, while a PVC is a request for storage by a user or service. The linkage between the two enables dynamic mounting of specific directories or datasets directly into containerized services, following a declarative provisioning model. Furthermore, using the SubPath capability, it is possible to mount a specific file or subdirectory from a volume into a container, rather than the volume’s root directory. Additionally, the use of StorageClass definitions allows administrators to define different backend types (e.g., CephFS, Cloud Storage, NFS, local-path, among other).

In this approach the *PrepareData* service acts by creating a PV/PVC pair that maps the requested RSE dataset to a dedicated mount path within the user’s home directory on the pod itself. At runtime, the corresponding Kubernetes deployment must be customized to recognize which PVCs are associated with the user. These PVCs must be mounted as volumes during pod instantiation, ensuring that the dataset is accessible within the user’s containerized session. This design pattern is effective for data delivery in orchestrated environments, but it introduces a coupling between *PrepareData* and the instantiation logic of the consuming service.

Similar to the mount bind approach, the use of PVs for data linking requires deployment-time awareness and injection of volume declarations, which must be dynamically associated with each user session by using a mapping database. This necessitates a certain degree of flexibility and automation in service deployment templates, especially for multi-user environments such as JupyterHub or CARTA, among others. An example of this integration is presented in the following section, where we describe a JupyterHub instance deployed within a Kubernetes cluster using *PrepareData*-generated PVCs by customising the instantiation procedure.

Figure 6 illustrates how the *PrepareData* service handles requests using a PV and PVC strategy. Similar to the mount --bind approach, a backend database is used to track metadata for each dataset preparation. In this case, the database also stores metadata for managing the lifecycle of dynamically created PV/PVC resources.

For each RSE dataset-user pair requested, a dedicated PV/PVC is generated via the Kubernetes API, and scoped to the appropriate namespace corresponding to the consuming service.

The PV/PVCs volumes remain available for reuse, allowing applications such as JupyterHub to dynamically discover and inject the correct PVs into user sessions. This is achieved by querying the backend database for active dataset preparations and attaching the volumes at runtime, or after a new session requested.

### 4.3 Integration scenarios for the SRCNet

The *PrepareData* service supports multiple integration strategies to tackle the diverse architectural and execution contexts within the SRCNet. Both implementations — mount --bind and Kubernetes-native PV and PVC— aim to minimise data duplication and facilitate transparent access to datasets, they differ in their system-level requirements and the degree of deployment customisation needed. Specifically, the mount --bind strategy is applicable to both standalone and Kubernetes-based services, whereas the PV/PVC-based model is exclusive to Kubernetes environments. The following sections detail the integration characteristics and requirements of each approach.

***4.3.1 Standalone environments***

Standalone environments refer to services and applications that operate outside Kubernetes orchestration, such as traditional batch systems or containerised tools running directly on host systems. For these environments, *PrepareData* uses the native Linux mount --bind functionality to expose datasets stored at the RSE directly into a user’s home directory or working area allowing services to access data as if it were locally present. This method is transparent to the consuming application. Consequently, no internal modification of the service logic is required, making it highly suitable for integration with resource managers such as SLURM or HTCondor. For example, a user job executed via SLURM can access a dataset located at /mnt/rucio/.../testing/84/1c/galactic_center.fits through a mount --bind to the user’s area (e.g., /home/user/testing/), enabling immediate usage within the job script without requiring explicit data copying or staging mechanisms. *PrepareData* performs the bind operation across all relevant compute nodes to ensure availability, and relies on system privileges (via daemon or privileged containers) to execute the mount consistently. This approach remains effective provided that the file system is shared or mirrored across nodes but do not require container-level isolation.

***4.3.2 Kubernetes-based services***

Kubernetes has become the standard orchestration layer for many SRCNet services. Consequently, *PrepareData* has implementations based on mount --bind with HostPropagation, and PV/PVC-based delivery. Both require service-level customisation at deployment time to ensure seamless integration of user datasets.

**Mount propagation**

To enable full isolation and user-level data exposure, for example, the JupyterHub deployment can be configured so that each user is assigned a dedicated directory under a shared mount point, typically with this template /mnt/users/<username>/. This allows for direct integration with externally managed data, and provides consistency across sessions and services such as *PrepareData.*

To achieve this, the JupyterHub Helm chart values.yaml must be configured to,

• a) mount the parent directory /mnt/users into the user container, b) dynamically assign /mnt/users/<username> as the container’s working home directory, and c) ensure mount propagation is enabled so that bind-mounted data becomes visible.

An example configuration for the singleuser section in values.yaml is shown below:

singleuser:
...
extraVolumes:
- name: home-mount

hostPath:
path: /mnt/users
type: Directory

extraVolumeMounts:
- name: home-mount

mountPath: /mnt/users
mountPropagation: HostToContainer

extraEnv:
NB_USER: “{username}”.
HOME: “/mnt/users/{username}”
lifecycleHooks:
postStart:
exec:
command:
- sh
- -c
- >
export HOME=/mnt/users/$(whoami) && cd $HOME



This configuration ensures that the environment variable $HOME is redirected to the correct location, matching the IAM-provided username. The user is automatically placed inside their designated folder upon session start.

In combination with *PrepareData*, any dataset bind-mounted into /mnt/users/<username>/<namespace>/becomes directly accessible within JupyterHub without requiring any additional volume provisioning, copying, or duplication.

**Dynamic injection of PVs/PVCs via spawner hooks in JupyterHub**

For services running under Kubernetes, the most native integration of user-specific datasets delivered by *PrepareData* is through dynamically created PVs/PVCs. In this workflow, each dataset requested by a user is exposed as a PV/PVC and recorded in a lightweight metadata store linked to the user’s IAM identity. When a new JupyterHub session is initiated, the KubeSpawner pre-spawn hook, a customizable function that runs just before the user’s container is launched and that enables the modification of the environment variables or the injection of PV/PVC. Therefore, this hook queries the metadata store, retrieves the relevant PVs/PVCs, and injects them into the pod specification. As a result, when the session is opened by the user datasets are seamlessly available within the container.

An example of the pre-spawn hook logic in Python is shown below:

...
def pre_spawn_hook(spawner):
username = spawner.user.name

pvc_list = query_user_pvcs_from_db(username)
for pvc in pvc_list:
spawner.volumes.append({
‘name’: pvc[‘name’],

‘persistentVolumeClaim’:
 {‘claimName’: pvc[‘name’]}
 })
spawner.volume_mounts.append({
‘mountPath’: pvc[‘mountPath’],

‘name’: pvc[‘name’]
 })



A corresponding JupyterHub configuration snippet to enable this hook is as follows:

c.KubeSpawner.pre_spawn_hook = pre_spawn_hook




For example, if a dataset is prepared as pvc-user-namespace-m31-fits, and designated to be mounted at /home/jovyan/<namespace>/, the user will see it immediately upon logging in.

## 5. Experimental results and operational trade-off analysis

The benchmarks were initiated from a Kubernetes cluster. The RSE and user area were backed by separate CephFS volumes exposed through the CSI stack. CephFS data transfer and storage I/O are directly exercised by the copy benchmark. By contrast, bind-mount creation primarily exercises local VFS and kernel namespace operations, whereas PV/PVC creation and binding primarily exercise Kubernetes control-plane and CSI orchestration. Consequently, the three experiments do not represent equivalent interactions with the storage backend.

The experiments characterise three distinct operational mechanisms used by PrepareData: (i) transferring a dataset from the RSE to the user area, (ii) creating Linux bind mounts that expose RSE-resident paths within a user-accessible namespace, and (iii) creating and binding PV/PVC pairs through the Kubernetes storage abstraction. These experiments measure different operations and are therefore analysed as separate operational cases rather than as equivalent performance alternatives.

To ensure the reproducibility of the benchmarks and results presented in this study, the following experimental environment and procedures were established:

• The evaluation was conducted on a Kubernetes (v1.28.2) cluster consisting of one control plane node and three worker nodes. Each node was provisioned with 8 vCPUs, 32 GB of RAM, and a 100 GB local root disk, running Ubuntu 22.04 LTS with its native kernel.
• The internal networking infrastructure provided a high-speed 100 Gbps interconnect between nodes, while external internet connectivity was constrained to a 10 Gbps bandwidth. Storage was managed via Ceph (version Reef 18.2.2 stable), utilizing two distinct CephFS volumes: one dedicated to the local Rucio Storage Element (RSE) and another for the user area. Both volumes were configured to be globally visible and mounted across all nodes in the cluster.
• All tests were performed using PrepareData version 0.1. The benchmarking suite is publicly available and can be retrieved from the Zenodo repository ^29^ or via the following repository: https://github.com/manuparra/preparedata-tests.git.

The testing framework evaluates three specific operational environments: copy, mount, and PV/PVC. To replicate these tests, the following prerequisites must be met:

• Administrative Access: The execution environment requires kubectl access with sufficient privileges to provision PVs/PVCs.
• Storage Visibility: Verified mounting capabilities and visibility of the CephFS-based RSE and user area across all cluster nodes.
• Execution: Following verification, the benchmarks are triggered through the execution of the scripts copy.sh, mount.sh, and pvpvc.sh.

The raw output generated by these scripts constitutes the data source for this analysis and the results tables detailed in this subsection.

For the copy experiment, the independent variable was the amount of data transferred, with file sizes ranging from 100 MB to 10240 MB. For the bind-mount experiment, sequential batches ranging from 10 to 2000 mount operations were created. For the PV/PVC experiment, sequential batches ranging from 10 to 1000 PV/PVC pairs were provisioned. All configurations were repeated 10 times.

The common wall-clock metric represents the time required to reach a mechanism-specific readiness condition. For copy-based delivery, readiness means that the complete payload has been transferred to the user area. For bind mounts, readiness means that all requested mount operations have completed successfully. For PV/PVC provisioning, readiness means that the requested claims have reached the Bound state. These conditions are operationally useful within each mechanism, but they are not semantically equivalent and must not be used to construct a direct performance ranking.

Table 1 summarises the average wall-clock time required to copy datasets of increasing size from the CephFS-backed RSE to the user area.

**Table 1.** Average wall-clock time required to complete a CephFS-to-CephFS dataset copy from the RSE to the user area. Values represent means over ten runs.

Size (MB)	Avg. Time (s)
100	0.131
200	0.206
500	0.429
1024	0.830
2048	1.510
3072	2.170
4096	2.830
5120	3.607
10240	7.213

Within the evaluated testbed, copy completion time increased approximately linearly with dataset size, corresponding to an effective transfer rate of approximately 1.3–1.4 GB/s for the larger files. This experiment characterises actual payload movement and CephFS read/write activity between the RSE and the user area. Its wall-clock values should not be compared numerically with bind-mount creation or PV/PVC binding times, because those experiments do not transfer an equivalent data payload. The results also do not characterise subsequent application-level access to the copied dataset. Operationally, the main trade-off of this approach is that every preparation creates an additional full copy and consumes storage capacity proportional to the requested dataset size.

Table 2 presents the results of the bind-mount setup experiment, in which sequential batches of 10–2000 mount operations were created. The reported wall-clock time represents the completion time for the entire batch and does not include subsequent application-level data access through the mounted paths.

**Table 2.** Bind-mount setup results. The columns report the sequential batch size, average batch-completion time, and system CPU fraction measured by the benchmark driver. Columns report the number of mount operations, the average wall-clock time, and the fraction of CPU time spent in system mode (kernel).

Number of Mounts	Avg. Time (s)	Sys CPU Fraction
10	0.109	0.082
50	0.391	0.109
100	0.773	0.115
200	1.571	0.112
400	3.563	0.108
800	9.227	0.114
1000	13.242	0.123
2000	43.834	0.129

Bind-mount batch-completion time increases approximately with the number of mount operations in the evaluated range. A batch of 100 mounts completed in less than one second, whereas a batch of 1,000 mounts required approximately 13 seconds. The system CPU fraction remained between approximately 0.10 and 0.13, indicating a relatively consistent amount of kernel-side work in the benchmark driver as the batch size increased. These measurements characterise only the cost of creating the filesystem namespace mappings. They do not measure read throughput, read latency, metadata performance, or application behaviour through the resulting mounted paths. Therefore, the short setup time observed for bind mounts must not be interpreted as evidence of superior end-to-end or application-level performance. Operationally, the mechanism may be suitable for services that can consume existing datasets through POSIX paths and for which filesystem-level exposure is compatible with the deployment and security model.

Table 3 reports the results of the static PV/PVC provisioning experiment. For each requested dataset, one PV and its corresponding PVC were created, and the benchmark waited until every claim reached the Bound state. The reported wall-clock time therefore represents Kubernetes object creation, controller reconciliation, claim binding, and associated CSI coordination rather than dataset transfer or subsequent application I/O.

**Table 3.** Static PV/PVC setup results. The columns report the number of PV/PVC pairs, average time until all claims reached the Bound state, and the system CPU fraction measured by the benchmark driver. Columns report the number of PV/PVC pairs, the average wall-clock time, and the system CPU fraction.

Number of PV/PVC	Avg. Time (s)	Sys CPU Fraction
10	5.106	0.315
50	25.403	0.318
100	50.757	0.312
200	104.217	0.312
400	206.714	0.310
800	413.254	0.312
1000	517.851	0.315

Within the evaluated testbed, the time required to create and bind PV/PVC pairs increased approximately with the number of Kubernetes objects. Batches of ten pairs reached the Bound state in approximately 5 seconds, whereas batches of 1000 pairs required approximately 518 seconds. These values characterise Kubernetes API, controller, and CSI orchestration overhead under the tested configuration. They are not directly comparable with bind-mount setup times or copy completion times, because the operations, readiness conditions, and independent variables differ. In particular, the longer initial setup observed for PV/PVC provisioning does not indicate lower read performance after attachment. Once a claim has been bound and mounted into a user session, the mechanism may remain operationally appropriate for Kubernetes-native services requiring storage lifecycle integration, isolation, or declarative attachment. Post-attachment application I/O was not evaluated in these experiments.

The experiments characterise different components of the PrepareData workflow and should be interpreted according to the operation performed by each mechanism. Copy-based delivery measures actual payload transfer between two CephFS-backed areas. Its completion time is driven primarily by dataset size and available storage throughput. Its principal operational trade-off is the creation of a complete additional copy, resulting in proportional storage consumption and repeated data movement when the same dataset is prepared for multiple users or sessions.

Bind-mount delivery does not transfer the underlying dataset during preparation. The experiment instead measures the cost of creating filesystem namespace mappings as the number of requested paths increases. This avoids a complete additional copy and may be appropriate for shared or read-mostly access patterns where POSIX path exposure is compatible with the consuming service. However, the experiment does not establish how applications perform when reading through those paths, and no conclusion about post-preparation throughput or latency can be derived from mount-creation time. PV/PVC delivery also avoids copying the complete dataset during the measured preparation stage, but its setup path is based on Kubernetes API objects, controller reconciliation, and CSI integration. The measured time until the claims reached the Bound state therefore represents orchestration overhead rather than data-transfer performance. The additional setup work may be acceptable for Kubernetes-native services that benefit from declarative attachment, isolation, lifecycle management, or integration with existing storage abstractions. No conclusion about application I/O after attachment can be drawn from the provisioning measurements.

A related Kubernetes-native alternative is the use of ephemeral volumes whose lifecycle is coupled to the user-session Pod. This approach may simplify cleanup and reduce the risk of long-lived orphaned storage resources. CSI ephemeral volume support depends on the capabilities of the underlying CSI driver, and its suitability for exposing existing RSE-resident datasets requires further investigation.

Taken together, the results do not identify a universally fastest or best-performing PrepareData mechanism. The reported wall-clock values correspond to different operations, different scaling variables, and different readiness conditions. They should therefore be used to understand mechanism-specific operational costs and architectural trade-offs, not to construct a general performance ranking. Selection should instead consider whether the target service requires data copying, shared POSIX exposure, Kubernetes-native lifecycle integration, isolation, service customisation, or support for standalone execution.

Non-copying mechanisms may require the consuming service or its deployment configuration to be adapted so that the exposed paths are resolved correctly. This constitutes an integration trade-off rather than a performance result. The suitability of a mechanism therefore depends not only on its preparation overhead, but also on the filesystem assumptions, isolation requirements, permissions model, and orchestration environment of the consuming service.

Table 4 summarises the operational characteristics of the PrepareData delivery mechanisms evaluated in the prototype SRCNet testbed. The table does not rank the mechanisms by performance. Instead, it identifies the operation measured for each approach, its readiness condition, dominant source of overhead, deployment scope, and whether subsequent application-level I/O was evaluated. The timing results remain mechanism-specific and testbed-specific.

**Table 4.** Operational trade-offs of the evaluated PrepareData delivery mechanisms. The timing values reported in Tables 1–3 measure different operations and are not directly comparable Table should be interpreted as guidance for selecting a mechanism according to local SRC and service requirements. It is not a direct performance comparison and does not establish a universal ordering among the approaches.

Approach	Operation measured	Scaling variable	Readiness condition	Dominant measured overhead	Full dataset copied	Deployment scope	Service adaptation	Post-preparation application I/O evaluated
Copy	CephFS-to-CephFS payload transfer	Dataset size	Complete payload available in the user area	Storage read/write throughput and data volume	Yes	Standalone and Kubernetes	Generally no	No
Bind mount	Creation of filesystem namespace mappings	Number of mount operations	All requested bind mounts completed	VFS, mount syscalls, and namespace handling	No	Standalone; Kubernetes when compatible mount propagation and privileges are available	Possibly	No
PV/PVC	Creation and binding of PV/PVC pairs	Number of Kubernetes objects	All requested PVCs reached the Bound state	API server, controllers, and CSI orchestration	No	Kubernetes	Usually yes	No

## 6. Conclusion and future work

This work has addressed the problem of delivering scientific datasets from local RSEs to user working areas within an infrastructure such as SRCNet. Making data operationally available across multiple SKA Regional Centres requires delivery mechanisms that accommodate different storage systems, execution environments, and service-integration models. Copy-based delivery remains a valid and straightforward baseline because it provides broad compatibility with user-facing services, particularly legacy and standalone applications. However, each preparation requires the complete dataset to be transferred to the user area, resulting in additional preparation time, proportional storage consumption, and repeated data movement when the same dataset is requested by multiple users or sessions. These observations characterise the preparation stage and do not, by themselves, describe subsequent application-level data-access performance.

To address the storage duplication and repeated data movement associated with copy-based delivery, this work has implemented two non-copying data-exposure mechanisms: Linux bind mounts and Kubernetes-native PV/PVC attachment. Both mechanisms expose an existing RSE-resident dataset without creating an additional full copy and can support shared or read-mostly access patterns. However, they follow fundamentally different preparation paths. Bind-mount setup primarily involves local VFS, mount, and namespace operations, whereas PV/PVC setup involves Kubernetes API objects, controller reconciliation, and CSI orchestration. Their measured preparation times are therefore not directly comparable and should not be interpreted as evidence of relative end-to-end or application-level performance.

The bind-mount strategy leverages native Linux kernel functionality and may be appropriate for standalone applications and cluster schedulers such as SLURM and HTCondor when the underlying storage is exposed through compatible POSIX paths and the deployment permits the required mount operations. The PV/PVC-based approach follows the Kubernetes-native storage model and may be appropriate for services that require declarative volume attachment, isolation, and integration with Kubernetes lifecycle management. Its integration with JupyterHub was demonstrated through PV/PVC attachment using spawner hooks, and the same integration pattern may be applied to other Kubernetes-hosted services with compatible storage requirements. The experiments evaluated only the setup stage of these mechanisms; they did not measure application read throughput, access latency, or I/O behaviour after the dataset became available.

Importantly, these approaches do not define a one-size-fits-all solution. The appropriate strategy depends on the characteristics of each SRC node, including its storage backend, orchestration platform, permissions model, user workload patterns, and service-integration requirements. Production deployment in a multi-tenant environment will require additional security and lifecycle controls. For bind mounts, these include privilege minimisation, path validation, namespace isolation, access revocation, stale-mount detection, and reliable unmounting and cleanup. For PV/PVC-based delivery, lifecycle policies will be required to identify and remove expired or orphaned Kubernetes resources and to apply policy-based retention and deletion.

From a broader perspective, the non-copying mechanisms are consistent with green-computing and sustainable data-management principles because they can reduce redundant data movement and avoid the storage consumption associated with creating additional full copies. However, energy consumption and environmental impact were not measured directly in this study. The sustainability benefits should therefore be regarded as a design motivation and a potential consequence of reduced duplication, rather than as an experimentally quantified result.

Within this context, we highlight several limitations of the study. First, this evaluation focuses on local data-delivery mechanisms within a single SRC context and does not assess cross-SRC scenarios or wide-area deployments. Second, performance results are derived from representative workloads and infrastructure configurations, which may not fully reflect the diversity of storage backends, network conditions and user behaviours across the entire SRCNet. Third, proposed approaches rely on assumptions about administrative control that may not hold in all operational environments, particularly those with strict security or multi-tenancy constraints.

Nevertheless, several areas remain open for future exploration. These include the implementation of intelligent caching mechanisms, integration with object storage systems (e.g., S3, Ceph RGW), security, isolation, and retention and deletion policies as well as the development of a unified metadata and access tracking layer to support fine-grained policy enforcement across SRCs. Additionally, a comprehensive performance evaluation of the PV/PVC approach under realistic SKA-scale workloads would provide further insights into its operational viability.

## Ethics and consent

Ethical approval and consent were not required.

## Data availability

SRCNet preparedata tests: software and data [v1.0.2]. https://doi.org/10.5281/zenodo.17772362

This project contains the following underlying data:

• data/copy_time.csv. Raw per-run timing measurements used to compute average copy performance. Table 1
• data/mount_benchmark.csv. Raw wall-clock time and CPU-usage values for mount–bind scalability experiments. Table 2.
• data/pvc_benchmark.csv. Raw timing and CPU metrics for PV/PVC provisioning tests. Table 3.

Data are available under the terms of the Creative Commons Zero “No rights reserved” data waiver (CC0 1.0 Public domain dedication). ^29^

No datasets used in this study are subject to restrictions, and no embargo applies. All materials are fully accessible for reuse and replication.

## Software availability

SRCNet preparedata software v1.0.2. https://doi.org/10.5281/zenodo.17772362

• All source code is also available in a public GitHub:
**GitHub:** https://github.com/manuparra/preparedata-tests

This repository is under the terms of an open licence.

## Acknowledgements

MP, SSE, JG, JS, MM, LD, EJ and LVM acknowledge financial support from the grant CEX2021–001131-S funded by MICIU/AEI/ 10.13039/501100011033 and from the grant TED2021-130231B-I00 funded by MICIU/AEI/ 10.13039/501100011033 and by the European Union NextGenerationEU/PRTR, acknowledges financial support from the grant PID2021-123930OB-C21, PID2024-155817OB-I00 funded by MICIU/AEI/ 10.13039/501100011033 and by ERDF/EU, and by the grant INFRA24023 (CSIC4SKA) funded by CSIC. MP, JG, SSE, LVM acknowledge support from the European Science Cluster of Astronomy and Particle Physics ESFRI Research Infrastructures project that has received funding from the European Union’s Horizon 2020 research and innovation program under Grant Agreement No. 824064. Prototype of an SRC (SPSRC) service and sup-port funded by the Ministerio de Ciencia, Innovación y Universidades (MICIU), by the Junta de Andalucía, by the European Regional Development Fund (ERDF) and by the European Union NextGenerationEU/PRTR. The SPSRC acknowledges financial support from the Agencia Estatal de Investigación (AEI) through the “Center of Excellence Severo-Ochoa” award to the Instituto de Astrofísica de Andalucía (IAA-CSIC) (SEV-2017-0709) and from the grant CEX2021–001131-S funded by MICIU/AEI/ 10.13039/501100011033.

## Footnotes

^1^
SKA Regional Centres Network: https://www.skao.int/en/science-users/119/ska-regional-centres

^2^
DESY: Deutsches Elektronen-Synchrotron.

^3^
FermiLab: Fermi National Accelerator Laboratory.

^4^
GK repository: https://gitlab.com/ska-telescope/src/src-dm/ska-src-dm-da-service-gatekeeper/

^5^
CANFAR is a science platform that integrates, among other things, a data visualisation environment, notebooks, and Linux desktops.

^6^
uvicorn: https://uvicorn.dev/

^7^
celery: https://docs.celeryq.dev/en/stable/

^8^
HostToContainer feature: https://kubernetes.io/docs/concepts/storage/volumes/#mount-propagation
