# Supplementary material for: Towards a sustainable astronomical data infrastructure: Optimising linking data from the Rucio datalake to the users areas within the SKA Regional Centres Network
Source: Open Res Eur. 2026 Aug 10;6:18. Originally published 2026 Jan 9. [Version 3] doi: 10.12688/openreseurope.22118.3 (PMC13231140; doi:10.12688/openreseurope.22118.3)
Supplement: Peer review history

# Reviewer report: Reviewer response for version 3

**DOI:** 10.21956/openreseurope.26858.r78896

**Published:** 2026-08-12

**Authors:** Alexandra Theodoropoulou

**Affiliations:** 1 University of Patras, Patras, Greece

The authors have already addressed my review comments successfully. I have nothing further to add.

Is the study design appropriate and does the work have academic merit?

Yes

Is the work clearly and accurately presented and does it cite the current literature?

Partly

If applicable, is the statistical analysis and its interpretation appropriate?

Not applicable

Are all the source data underlying the results available to ensure full reproducibility?

Partly

Are the conclusions drawn adequately supported by the results?

Partly

Are sufficient details of methods and analysis provided to allow replication by others?

Partly

Reviewer Expertise:

ICT, Management Information Systems, Decision Support Systems, Business Analytics, Data Analytics, Digital Transformation

I confirm that I have read this submission and believe that I have an appropriate level of expertise to confirm that it is of an acceptable scientific standard.

# Reviewer report: Reviewer response for version 2

**DOI:** 10.21956/openreseurope.25562.r73826

**Published:** 2026-06-02

**Authors:** Richard Gebler

**Affiliations:** 1 Technische Universitat Dresden, Dresden, Saxony, Germany

I would like to thank the authors for the revised manuscript. I found the work very interesting, especially from a data engineering and scientific infrastructure perspective. The data volumes targeted by SKA/SRCNet are far beyond the scale of many conventional data platforms, and this makes the problem addressed in the paper highly relevant. In particular, I appreciated the focus on avoiding unnecessary data copies while still making large datasets usable from practical analysis environments.

 The manuscript presents PrepareData as a local data-delivery service that bridges Rucio-managed storage and user-facing workspaces. The core problem is that data stored in Rucio Storage Elements are not automatically visible or usable from tools such as JupyterHub, CARTA, standalone services, or HPC-like workflows. The paper compares three practical approaches for exposing these datasets: traditional copy-based delivery, Linux bind mounts / mount propagation, and Kubernetes-native PV/PVC-based delivery.

 From my perspective, the study has clear academic and practical merit. The topic is relevant, the architecture is concrete, and the implementation is useful for the community working on large-scale scientific data infrastructure. However, I would still answer some of the review questions with “partly”, because several aspects should be clarified or better framed.

 The work is mostly clearly presented, but the reference list still needs careful checking. I noticed at least one duplicate reference: the EOS paper appears twice in the bibliography. This should be corrected, and the authors should ensure that all in-text citations point to the intended references. This is not a fundamental scientific weakness, but it should be fixed before final acceptance.

 The main technical point concerns the benchmark framing. The comparison between copy-based delivery, bind mounts, and PV/PVC provisioning is useful, but these approaches do not measure the same type of operation. Copy-based delivery measures actual data movement and storage I/O. Bind mounts mainly measure filesystem/kernel namespace operations. PV/PVC provisioning measures Kubernetes control-plane and storage orchestration overhead. Therefore, “time until prepared” is a useful operational metric, but it is not sufficient as a direct performance comparison between the approaches.

 For example, a bind mount can be created very quickly, but this does not by itself show how well the data can subsequently be read by real applications. Similarly, a Kubernetes-based approach may have higher initial setup overhead, but once the volume is attached to a user session, it may still be operationally suitable for Kubernetes-native services. The manuscript should therefore continue to present the results as a trade-off analysis rather than as a general performance ranking.

 I would also suggest that the authors discuss additional access patterns in the outlook. Not every use case necessarily requires exposing complete datasets through a filesystem path. Depending on the scientific workflow, API-based access, subsetting services, object-storage-native access, pre-signed URLs, or services that deliver only the requested data region may be relevant. This does not mean that these alternatives are automatically better, but they would help position PrepareData within a broader modern data infrastructure landscape.

 A related Kubernetes-native alternative that should be mentioned is CSI Ephemeral Volumes. The current PV/PVC-based approach is valid and Kubernetes-native, but it may become operationally heavy if many users, datasets, or sessions lead to a large number of persistent Kubernetes storage objects. For transient user sessions, such as JupyterHub notebooks or short-lived analysis services, ephemeral volumes could be worth considering because the volume lifecycle is more tightly coupled to the pod lifecycle. This may reduce cleanup complexity and avoid long-lived orphaned PV/PVC resources. I do not think this needs to be implemented in the current paper, but it should be discussed as a relevant future direction.

 Finally, the security and lifecycle implications should be stated clearly as future work. Bind mounts require careful handling of privileges, isolation, path validation, revocation, stale mounts, and cleanup. PV/PVC-based approaches also require lifecycle management for orphaned resources and policy-based deletion. These are not necessarily weaknesses of the prototype, but they are important for production use in a multi-tenant scientific platform.

 Overall, I consider the manuscript valuable and suitable for acceptance with reservations / minor revisions. The remaining issues do not invalidate the contribution, but they should be addressed to avoid over-interpreting the benchmark results and to improve the clarity, reproducibility, and operational framing of the work.

Is the study design appropriate and does the work have academic merit?

Yes

Is the work clearly and accurately presented and does it cite the current literature?

Yes

If applicable, is the statistical analysis and its interpretation appropriate?

Not applicable

Are all the source data underlying the results available to ensure full reproducibility?

Partly

Are the conclusions drawn adequately supported by the results?

Partly

Are sufficient details of methods and analysis provided to allow replication by others?

Partly

Reviewer Expertise:

data engineering / infrastructure

I confirm that I have read this submission and believe that I have an appropriate level of expertise to confirm that it is of an acceptable scientific standard, however I have significant reservations, as outlined above.

# Response

**Published:** 2026-08-03

**Authors:** Manuel Parra Royón

**Affiliations:** Instituto de Astrofísica de Andalucía, Consejo Superior de Investigaciones Cientificas, Granada, Granada, Spain

We sincerely thank the reviewer for the careful assessment and constructive comments, which have significantly improved the clarity and rigor of the manuscript. The revised version now more explicitly defines the scope of the work and avoids any implication of direct performance comparison between mechanisms with fundamentally different operations.

 We have carefully reviewed and corrected the bibliography to ensure consistency between in-text citations and reference entries, removing duplicates, fixing incorrect links, and reordering entries where necessary. Regarding the evaluation framing, we fully agree that copy-based delivery, bind mounts, and PV/PVC provisioning are not directly comparable.

 The manuscript, introduction, results and conclusions have been revised throughout to clarify that each mechanism is evaluated in terms of its own preparation workflow: data transfer for copy-based delivery, kernel and filesystem setup for bind mounts, and Kubernetes orchestration overhead for PV/PVC provisioning. We now explicitly state that the reported wall-clock times reflect readiness under different semantics and should not be interpreted as a performance ranking. The results are therefore presented as a characterization of operational trade-offs rather than a comparative benchmark, and we also clarify that application-level performance (e.g., read throughput or end-to-end workflow latency) is out of scope.

 We have also mentioned the discussion of data-access models to acknowledge that filesystem-based delivery is only one approach among several modern alternatives, such as API-based access, subsetting services, and object-storage-native mechanisms. These are now positioned as complementary approaches and potential future extensions of PrepareData, rather than alternatives evaluated in this work. In addition, we have incorporated  CSI Ephemeral Volumes as a relevant Kubernetes-native in short-lived interactive environments. We clarify that this approach was not implemented or evaluated.

 Finally, we have strengthened the discussion on security and lifecycle management, highlighting operational requirements such as isolation, cleanup, and resource tracking for both bind-mount and PV/PVC-based approaches. We again thank the reviewer for this feedback, which has helped us improve the precision, scope definition, and overall robustness of the manuscript.

# Reviewer report: Reviewer response for version 2

**DOI:** 10.21956/openreseurope.25562.r72766

**Published:** 2026-04-25

**Authors:** Alexandra Theodoropoulou

**Affiliations:** 1 University of Patras, Patras, Greece

The revised manuscript has fully addressed my previous comments and can now be accepted for indexing.

Is the study design appropriate and does the work have academic merit?

Yes

Is the work clearly and accurately presented and does it cite the current literature?

Partly

If applicable, is the statistical analysis and its interpretation appropriate?

Not applicable

Are all the source data underlying the results available to ensure full reproducibility?

Partly

Are the conclusions drawn adequately supported by the results?

Partly

Are sufficient details of methods and analysis provided to allow replication by others?

Partly

Reviewer Expertise:

ICT, Management Information Systems, Decision Support Systems, Business Analytics, Data Analytics, Digital Transformation

I confirm that I have read this submission and believe that I have an appropriate level of expertise to confirm that it is of an acceptable scientific standard.

# Reviewer report: Reviewer response for version 1

**DOI:** 10.21956/openreseurope.23938.r70163

**Published:** 2026-03-20

**Authors:** Rana Veer Samara Sihman Bharattej Rupavath

**Affiliations:** 1 National Louis University, Chicago, Illinois, USA

The article presents **PrepareData**, a data-delivery service designed for the **SKA Regional Centre Network (SRCNet)** to make datasets stored in **Rucio Storage Elements (RSEs)** accessible within users’ working environments without repeatedly copying large files. The work proposes and evaluates two non-copy approaches alongside the copy baseline:

- A **filesystem-based approach** using Linux mechanisms (e.g., **bind mounts**, and/or symbolic linking concepts) to “expose” data into user workspaces.
- A **Kubernetes-based approach** using **Persistent Volumes (PVs)** and **Persistent Volume Claims (PVCs)** to inject datasets into user environments dynamically.

The paper describes architectural integration (e.g., via a gateway component for authorization) and reports performance experiments comparing these strategies in a prototype environment, emphasizing scalability, reduced duplication, and sustainability/“green computing” considerations.

 2) Is the work clearly and accurately presented, and does it cite the current literature? **Partly**

**What works well**

- The core problem is clearly motivated: enabling access to very large datasets in distributed infrastructure without wasteful repeated copying.
- The paper provides useful architectural/workflow figures and concrete implementation detail (not purely conceptual).

**What needs improvement (must address)**

- **References appear inconsistent/incomplete in format** in places (per the review text), and some citations may lack basic bibliographic information.
- The reviewer flags that **some references are preprints (arXiv) rather than peer-reviewed sources and** suggests replacing them where possible with peer-reviewed, published literature.

**How authors can address**

- Standardize reference formatting (journal, year, volume/issue, pages, DOI where available).
- Replace arXiv/preprints with peer-reviewed versions when they exist; if none exist, justify why the preprint is used and ensure complete metadata.

3) Is the study design appropriate and does the work have academic merit? **Yes**

**Strengths**

- The study tackles a real, relevant systems problem in scientific data infrastructure.
- The solution is practical and aligns with established “move compute to data” concerns and scalable data-management needs.
- The architecture and implementation are described in enough detail to be useful to practitioners.

**Optional strengthening**

- Make the contribution claims explicit (see next section).

4) Are sufficient details of methods and analysis provided to allow replication by others? **Partly**

**What is present**

- Implementation details: service implemented in Python (FastAPI), asynchronous processing via Celery, API described, integration patterns described.
- Performance experiments are described (copy timing vs dataset size; “storms” of mount operations and PV/PVC provisioning), including hardware/software context (e.g., Kubernetes version, CephFS backing).

**What needs improvement (must address)**

- The reviewer indicates the manuscript needs **a clearer, explicit objectives/contributions section**, preferably near the end of the Introduction.
- The reviewer also suggests **limitations must be clearly presented**, rather than implied—this affects how replicable and scoped the results appear.

**How authors can address**

- Add a short subsection: **“Objectives and Contributions”** (what exactly is evaluated; what is claimed).
- Add a subsection near the end (before Conclusions or within it): **“Limitations”** (prototype constraints, single-node setup, what was not evaluated such as multi-site behavior, security enforcement validation, lifecycle enforcement in practice, etc.).
- Include a replication checklist: versions, configuration parameters, key scripts/commands, and where to find them (if not already clearly consolidated).

5) Statistical analysis and interpretation: **Not applicable**

 The work is primarily systems/performance benchmarking; the selected response in the document is “Not applicable.”

 (If the venue expects statistical treatment: authors could still add uncertainty measures - e.g., variance/CI across repeated runs - but that would go beyond what the reviewer marked.)

 6) Are all the source data underlying the results available to ensure full reproducibility? **Partly**

**What is present**

- A **Data availability** section exists and points to a **Zenodo deposit** that includes raw data files (e.g., CSVs for copy time, mount benchmarks, PVC benchmarks).
- A **Software availability** section exists, also pointing to Zenodo and a public GitHub repository.

**Why only “Partly” (likely)**

- Even with data and code shared, full reproducibility often also requires clear environment/configuration capture (cluster settings, CephFS/CSI config, resource limits, etc.). The reviewer’s “Partly” suggests something is missing or not fully transparent.

**How authors can address (must address)**

- Add a “Reproducibility” paragraph listing:
  - exact versions (Kubernetes, CSI driver, CephFS, OS/kernel),
  - cluster topology (nodes, CPU/RAM, network assumptions),
  - key configuration settings and how to run the benchmarks end-to-end.
- Ensure the repository/Zenodo package contains (or links to) scripts that reproduce tables/figures directly from raw data.

7) Are the conclusions adequately supported by the results? **Partly**

**Main issue (must address)**

- The review explicitly notes that the **performance comparison wording (especially Table 4)** risks sounding more definitive/universal than the experimental setup supports.
- The tests evaluate different overhead types (copy time vs mount operations vs control-plane provisioning latency), so they are **not strictly apples-to-apples**; the paper itself even notes they are “not directly comparable,” but the summary framing may still read like a universal ranking.

**How authors can address**

- Reframe conclusions to emphasize **trade-offs and context-specific suitability**, not “winner” claims.
- Adjust Table 4 wording (and surrounding text) to avoid universal performance rankings; tie each method to its intended operational context and what exactly was measured.
- Explicitly state what is demonstrated (prototype performance trends) and what is not (full production-scale multi-site evaluation).

Points that must be addressed to make the article scientifically sound (per the review’s reservations)

1. **Add an explicit statement of objectives and main contributions** (near end of Introduction).
2. **Add a clear limitations section** (before/within Conclusions).
3. **Fix and standardize references**; ensure complete bibliographic info and replace preprints with peer-reviewed sources where possible.
4. **Clarify the performance comparison framing** (especially Table 4 and related wording) so it does not read as a universal ranking beyond the scope of the experiments.
5. **Improve reproducibility documentation** beyond sharing data/code (environment/config details and clear “how to reproduce” instructions).

Is the study design appropriate and does the work have academic merit?

Yes

Is the work clearly and accurately presented and does it cite the current literature?

Partly

If applicable, is the statistical analysis and its interpretation appropriate?

Not applicable

Are all the source data underlying the results available to ensure full reproducibility?

Partly

Are the conclusions drawn adequately supported by the results?

Partly

Are sufficient details of methods and analysis provided to allow replication by others?

Partly

Reviewer Expertise:

ICT; Management Information Systems; Decision Support Systems; Business Analytics; Data Analytics; Digital Transformation.

I confirm that I have read this submission and believe that I have an appropriate level of expertise to confirm that it is of an acceptable scientific standard, however I have significant reservations, as outlined above.

# Response

**Published:** 2026-04-06

**Authors:** Manuel Parra Royón

**Affiliations:** Instituto de Astrofísica de Andalucía, Consejo Superior de Investigaciones Cientificas, Granada, Granada, Spain

We sincerely thank the reviewer for the detailed and constructive comments, which have helped us improve the clarity, methodological transparency, and reproducibility of the manuscript. In response to the comments, all bibliographic references have been carefully revised and updated to follow a more homogeneous citation style, including, whenever possible, the complete metadata requested. In addition, all previous references to arXiv preprints have been replaced by their corresponding peer-reviewed journal articles or conference proceedings whenever published versions were available. We have added a new paragraph at the end of the Introduction explicitly clarifying the main objectives of the study and its concrete scientific and technical contributions. Likewise, an additional paragraph has been incorporated into the Conclusions to explicitly discuss the limitations of the current work, making it easier for readers to understand how these limitations may affect both the reproducibility of the proposed approach and the interpretation of the reported results. Several new paragraphs have been incorporated into the Results section describing in detail the experimental and deployment environment required to reproduce the tests. This environment is now fully specified to facilitate complete reproducibility of the reported experiments, including a list of the required components and configuration items needed to deploy and execute the tests. Additionally, the outputs generated by the testing framework, which are publicly available through Zenodo and the associated GitHub repository, are directly exported in CSV format, corresponding to the data included in the manuscript. The Zenodo record itself also provides the execution instructions required to reproduce the test workflow. We have introduced an explicit clarification stating that the comparison presented in Table 4 should not be interpreted as a universal ranking of approaches, but rather as a qualitative and context-dependent evaluation performed within a specific testbed scenario. Finally, to reinforce the scope and current maturity of the work, the Conclusions now include an additional paragraph explicitly describing the type of development represented by this contribution and its present limitations, helping delimit the current applicability of the proposed solution.

# Reviewer report: Reviewer response for version 1

**DOI:** 10.21956/openreseurope.23938.r70476

**Published:** 2026-03-12

**Authors:** Alexandra Theodoropoulou

**Affiliations:** 1 University of Patras, Patras, Greece

This is a real technical contribution with practical value for SRCNet and the paper is not weak in concept. The core problem is well motivated, namely how to expose RSE-resident data to user workspaces without repeated copying in a heterogeneous environment of standalone and Kubernetes-based services. The architecture and workflow figures are also useful and the paper gives concrete implementation detail rather than staying abstract. That said, the manuscript still needs certain revisions before final indexing.

 The paper needs a brief, explicit paragraph, preferably near the end of the Introduction, that clearly states the study objectives and the main contributions of the paper, instead of mentioning them implicitly and in a narrative form. The same goes for the limitations of the study; they need to be clearly presented, preferably at the end of the paper (before Conclusions or as a subsection in Conclusions.)

 One point that needs some further clarification is the way the performance comparison is presented, especially in Table 4, because the current wording can sound broader and more definitive than the actual evaluation setup supports. This does not mean that the reported results are invalid; rather, the concern is that the three approaches were tested under a limited prototype environment and do not measure exactly the same type of overhead, so they should not read as a universal ranking. A better way to present this would be to soften the comparative wording and explain more clearly that the findings reflect the specific testbed and mainly show different trade-offs and practical use cases, rather than one method being generally superior in all settings.

 The discussion of access control and operational safeguards would benefit from clearer wording, because some parts of the manuscript may give the impression that these aspects were fully validated in practice, while they are mainly presented at the architectural level. This does not mean that the design is not sound; the concern is simply that the current evaluation focuses on performance and deployment behavior rather than on security enforcement, revocation or lifecycle control. A clearer presentation would be to state more explicitly that these features are part of the proposed system design and intended management model, while the present experiments demonstrate functional integration and performance under the tested setup.

 The references seem inconsistent in their format, lacking basic information. Also, there are a few preprints/papers that have not been peer-reviewed (arXiv database). Authors need to find all the information required for their references (e.g. ref no22) and replace the preprints with peer-reviewed, published papers.

Is the study design appropriate and does the work have academic merit?

Yes

Is the work clearly and accurately presented and does it cite the current literature?

Partly

If applicable, is the statistical analysis and its interpretation appropriate?

Not applicable

Are all the source data underlying the results available to ensure full reproducibility?

Partly

Are the conclusions drawn adequately supported by the results?

Partly

Are sufficient details of methods and analysis provided to allow replication by others?

Partly

Reviewer Expertise:

ICT, Management Information Systems, Decision Support Systems, Business Analytics, Data Analytics, Digital Transformation

I confirm that I have read this submission and believe that I have an appropriate level of expertise to confirm that it is of an acceptable scientific standard, however I have significant reservations, as outlined above.

# Response

**Published:** 2026-04-06

**Authors:** Manuel Parra Royón

**Affiliations:** Instituto de Astrofísica de Andalucía, Consejo Superior de Investigaciones Cientificas, Granada, Granada, Spain

We sincerely thank the reviewer for the careful reading of the manuscript and for the constructive observations provided. Following these comments, we have improved the wording surrounding the context of Table 4, explicitly reinforcing that the comparison does not address security enforcement, revocation mechanisms, or lifecycle control aspects, but is instead focused on the performance behaviour of each preparation flavor under the specific experimental conditions considered. The paragraph preceding Table 4 has been revised to clearly state that the intention is not to establish that one study or approach is universally better than another, but rather to provide a quantitative and context-dependent comparison within the specific testbed used in this work. In addition, we have strengthened the Introduction by adding an explicit paragraph describing the main objectives and the specific contributions of the work. Likewise, the Conclusions section now includes a dedicated discussion of the current limitations of the study. These additions clarify that the main scope of the manuscript is centred on the design, deployment, and performance evaluation of the proposed solutions, rather than on the full lifecycle management of prepared datasets or their associated security models. Finally, all bibliographic references have been carefully revised and updated to follow a more homogeneous citation format, including, whenever possible, the complete metadata requested. Furthermore, all citations previously referring to arXiv preprints have been replaced by their corresponding peer-reviewed journal articles or conference proceedings whenever published versions were available.
